# Construction of a radiomics model based on CT imaging for predicting capsular invasion in thymomas

**DOI:** 10.3389/fradi.2025.1707488

**Published:** 2025-12-12

**Authors:** Shuo Liang, Yanhong Chen, Jianhui Li, Zhenchun Song, Li Zhou, Rui Yin

**Affiliations:** Department of Radiology, Tianjin Chest Hospital, Tianjin, China

**Keywords:** thymoma, radiomics, x-ray computed tomography, machine learning, capsular invasion

## Abstract

**Objective:**

To develop a radiomics-based predictive model for capsular invasion in thymomas by applying machine learning algorithms to non-contrast and contrast-enhanced CT imaging. This study aimed to assess the influence of intratumoural and peritumoural regions on capsular invasion prediction and to compare the performance of models derived from these regions within the same dataset, thereby identifying the optimal predictive model.

**Methods:**

Clinical and imaging data were retrospectively collected from 151 patients with thymoma who underwent treatment at Tianjin Chest Hospital between June 2018 and January 2025. Based on pathological findings, patients were categorised into capsular invasion and non-invasion groups and subsequently randomised into a training set (*n* = 106) and a test set (*n* = 45) in a 7:3 ratio. Radiomic feature selection was performed using univariate logistic regression analysis followed by least absolute shrinkage and selection operator (LASSO) regression. Predictive models were developed employing multiple machine learning algorithms, including logistic regression. Model performance was evaluated through receiver operating characteristic (ROC) curve analysis, with sensitivity, specificity, F1 score, and decision curve analysis (DCA) used to assess diagnostic accuracy and clinical applicability. DeLong's test was applied to compare the area under the curve (AUC) values between different models. Calibration curves were generated to evaluate model calibration, and model interpretability was examined using the Shapley Additive exPlanations (SHAP) method.

**Results:**

Comparative analysis of machine learning methods across different tumour regions revealed that the support vector machine (SVM) model, developed using radiomic features from the 4 mm peritumoural region on contrast-enhanced CT scans, demonstrated optimal predictive performance. This model achieved area under the curve (AUC) values of 0.890 [95% confidence interval (CI): 0.823–0.956] in the training cohort and 0.888 (95% CI: 0.792–0.983) in the test cohort.

**Conclusion:**

CT-based radiomics demonstrates efficacy in predicting capsular invasion in thymomas, with the peritumoural region proving particularly significant. This methodology shows potential for supporting clinicians in preoperative treatment strategy formulation.

## 1 Introduction

Thymoma represents the most prevalent primary neoplasm of the anterior mediastinum (1). The widespread adoption of CT screening has led to an increasing incidence of incidental thymoma diagnoses in asymptomatic patients. Surgical resection constitutes the primary therapeutic intervention, with both surgical approach selection and prognostic assessment dependent on the Masaoka-Koga staging system (2), Stage I thymomas, characterised by an intact tumour capsule, demonstrate an R0 resection rate and 5-year survival rate approaching 100%, with an associated recurrence rate of approximately 0.9% (3), Such cases typically do not require postoperative radiotherapy. Conversely, stage IIb and advanced thymomas displaying capsular invasion necessitate adjuvant radiotherapy and are associated with significantly elevated recurrence risks (4). Current confirmation of capsular invasion relies exclusively on postoperative histopathological examination. While contrast-enhanced CT serves as the primary non-invasive imaging technique for thymoma evaluation, its interpretation remains subject to radiologists' subjective assessment, resulting in considerable interobserver variability and experience-dependent outcomes. Consequently, conventional CT imaging demonstrates limited accuracy in precisely evaluating thymoma invasiveness, thereby restricting clinicians' ability to develop optimal treatment strategies. This underscores the critical unmet clinical need for reliable preoperative non-invasive prediction of capsular invasion.

Emerging evidence suggests that the tumour microenvironment plays a pivotal role in tumour invasiveness and prognosis (5). Radiomics, an advancing field of translational research, utilises artificial intelligence to analyse texture features and extract substantial latent information from conventional medical images (6). This approach facilitates non-invasive characterisation of tumour heterogeneity, providing considerable clinical advantages for disease diagnosis and outcome prediction. Consequently, radiomics has found widespread application in oncological diagnosis, tumour staging, and prognostication (7).

**Figure 1:**
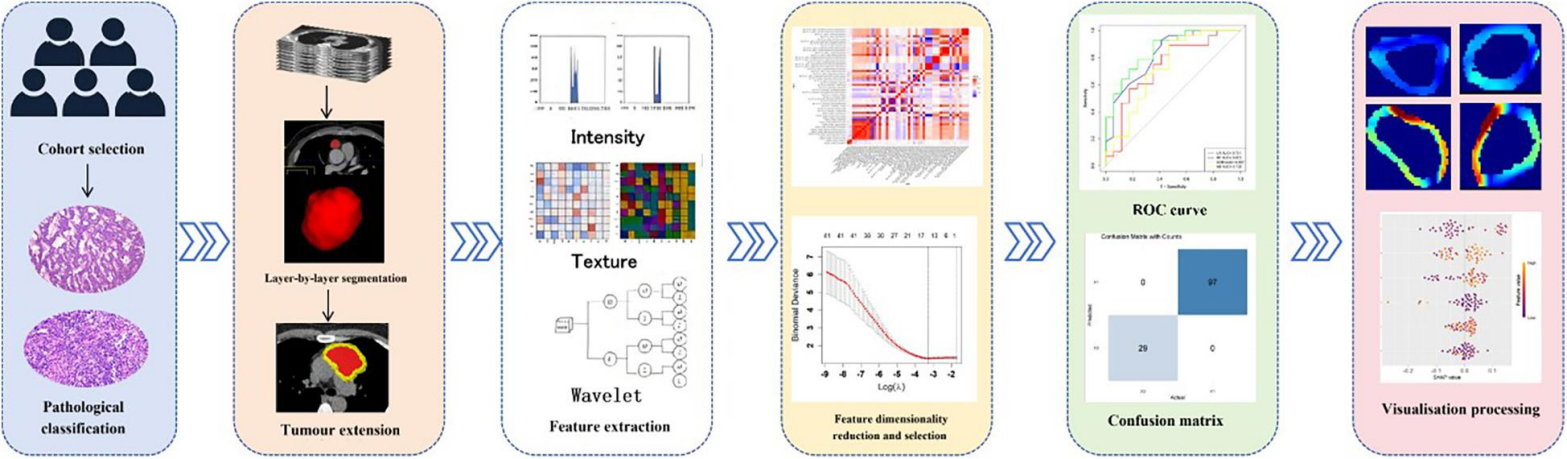
Study workflow diagram.

This study proposes to develop machine learning models for predicting thymoma capsular invasion through extraction of radiomic features from both intratumoural and peritumoural regions in CT imaging. A comprehensive comparison will be conducted to evaluate the predictive performance of different models and identify the optimal one with highest predictive accuracy. Furthermore, model interpretability will be enhanced by implementing Shapley Additive Explanations (SHAP) analysis (Figure 1).

## 2 Materials and methods

### 2.1 General data

A retrospective analysis was performed on 218 thymoma patients treated at Tianjin Chest Hospital from June 2018 to January 2025. The inclusion criteria comprised: (1) histopathological confirmation of thymoma post-surgical resection; (2) no preoperative treatment; (3) availability of high-quality CT images free from respiratory motion artefacts. Exclusion criteria were: (1) missing preoperative CT imaging, absence of surgical resection, or incomplete clinical data including pathological results; (2) suboptimal image quality; (3) presence of multiple thymic lesions (≥2 lesions).

**Figure 2:**
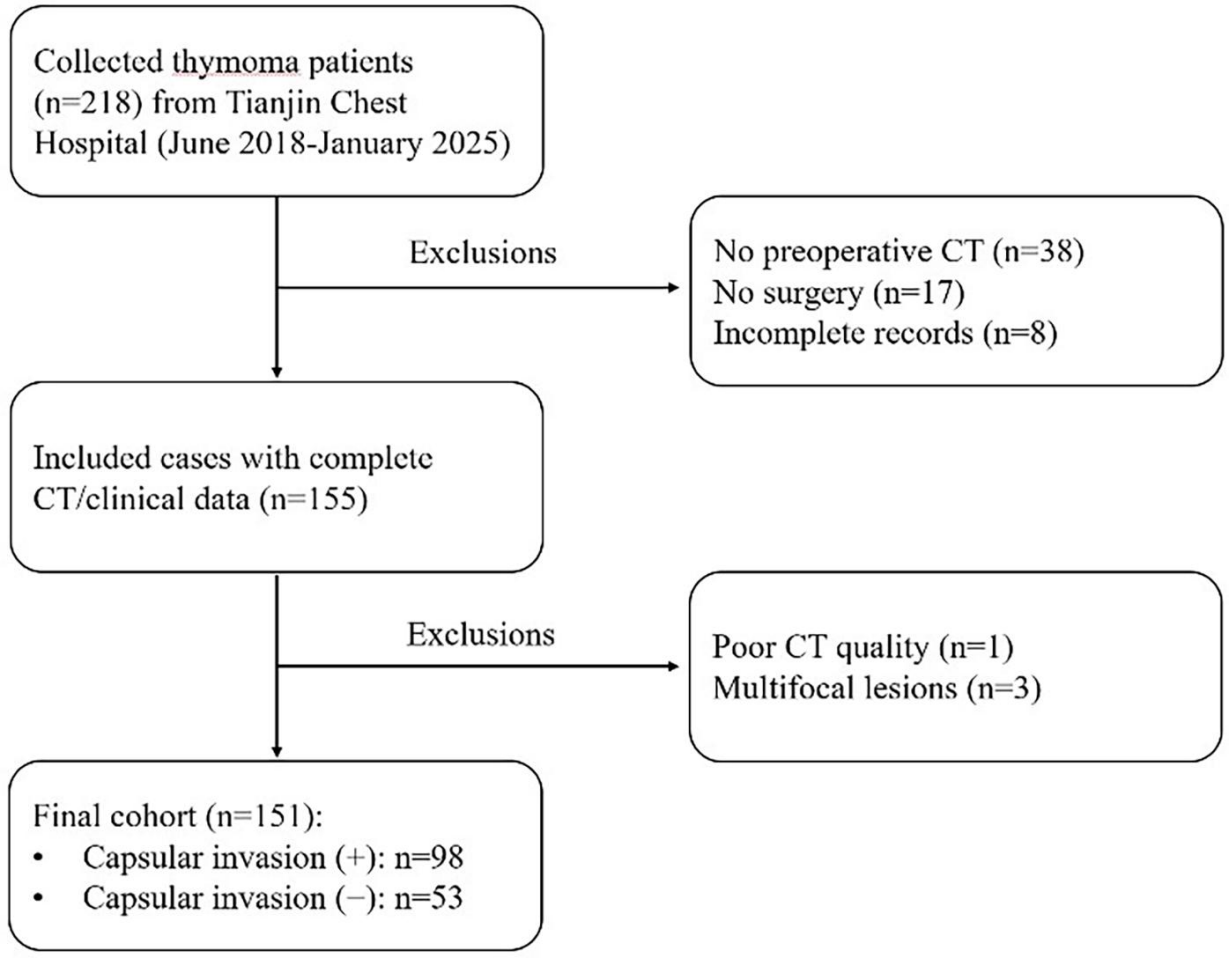
Patient enrolment flowchart.

The exclusion criteria resulted in the removal of 38 patients without preoperative CT imaging data, 17 patients who did not undergo surgical resection, and 8 patients with incomplete clinical data, leaving 155 patients with complete CT and clinical records for initial inclusion. Subsequent review of CT images led to the further exclusion of 3 patients with multiple lesions and 1 patient with inadequate image quality. The final study cohort therefore consisted of 151 patients, including 98 with pathologically confirmed capsular invasion and 53 without capsular invasion (Figure 2). For all enrolled patients, clinical data including sex, age, clinical symptoms and comorbidities were systematically recorded. Histopathological evaluation was performed on surgically resected specimens through standard processing involving formalin fixation, paraffin embedding, microtome sectioning, and haematoxylin and eosin (H&E) staining.

This study was conducted in accordance with the principles of the Declaration of Helsinki. The research protocol was reviewed and approved by the hospital ethics committee (Approval No. 2025 Ethics Review 21). Informed consent was obtained from all participants prior to inclusion in the study. All patient identifiers were removed, and the data were fully anonymized before analysis to ensure confidentiality and privacy.

### 2.2 CT scanning protocol

The CT examinations were performed using either a GE Revolution CT or Siemens SOMATOM Force scanner (Table 1).

**Table 1:** CT scanning protocol.

Equipment name	Scan range	Tube voltage	Tube current	Scan slice thickness	Reconstruction slice thickness	Reconstruction interval	Scan matrix	Gantry rotation time	Pitch	Reconstruction algorithm
GE Revolution CT	from the thoracic inlet to the diaphragm	120 kV	automated intelligent mA (Auto mA)	5.0 mm	1.0 mm	1.0 mm	512 × 512	0.6s	0.992	ASIR-V 30%
Siemens SOMATOM Force CT		120 kV	CARE Dose4D mode for tube current modulation	10.0 mm	1.5 mm	1.5 mm	512 × 512	0.5s	0.6	FBP convolution kernel Br40

Contrast Agent Administration Protocol: Patients fasted for 4 to 6 h before the examination. Intravenous administration of 80 to 90 mL ioversol (Visipaque™ 320 mgI/mL, GE Healthcare) was performed through the antecubital vein at an injection rate of 3.5 mL/s. Arterial phase imaging commenced 30 S following contrast agent injection.

### 2.3 Image quality assessment

Preoperative contrast-enhanced CT images from 152 patients with complete lesion data were independently evaluated by two radiologists, with 10 and 1 year of diagnostic imaging experience respectively. The subjective image quality assessment employed a 5-point grading scale: 5 indicated excellent quality with no significant noise or artefacts, optimal visualisation of anatomical structures and lesions, and fully suitable for definitive assessment; 4 represented good quality with mild noise or artefacts, reasonably clear delineation of anatomical structures and lesions, and adequate for assessment; 3 denoted moderate quality with noticeable noise or artefacts where most anatomical structures and lesions remained diagnostically interpretable; 2 corresponded to poor quality with severe noise or artefacts resulting in inadequate delineation of anatomical structures and lesions, rendering most regions unsuitable for diagnosis; 1 was classified as non-diagnostic.

### 2.4 Image segmentation and feature extraction

**Figure 3:**
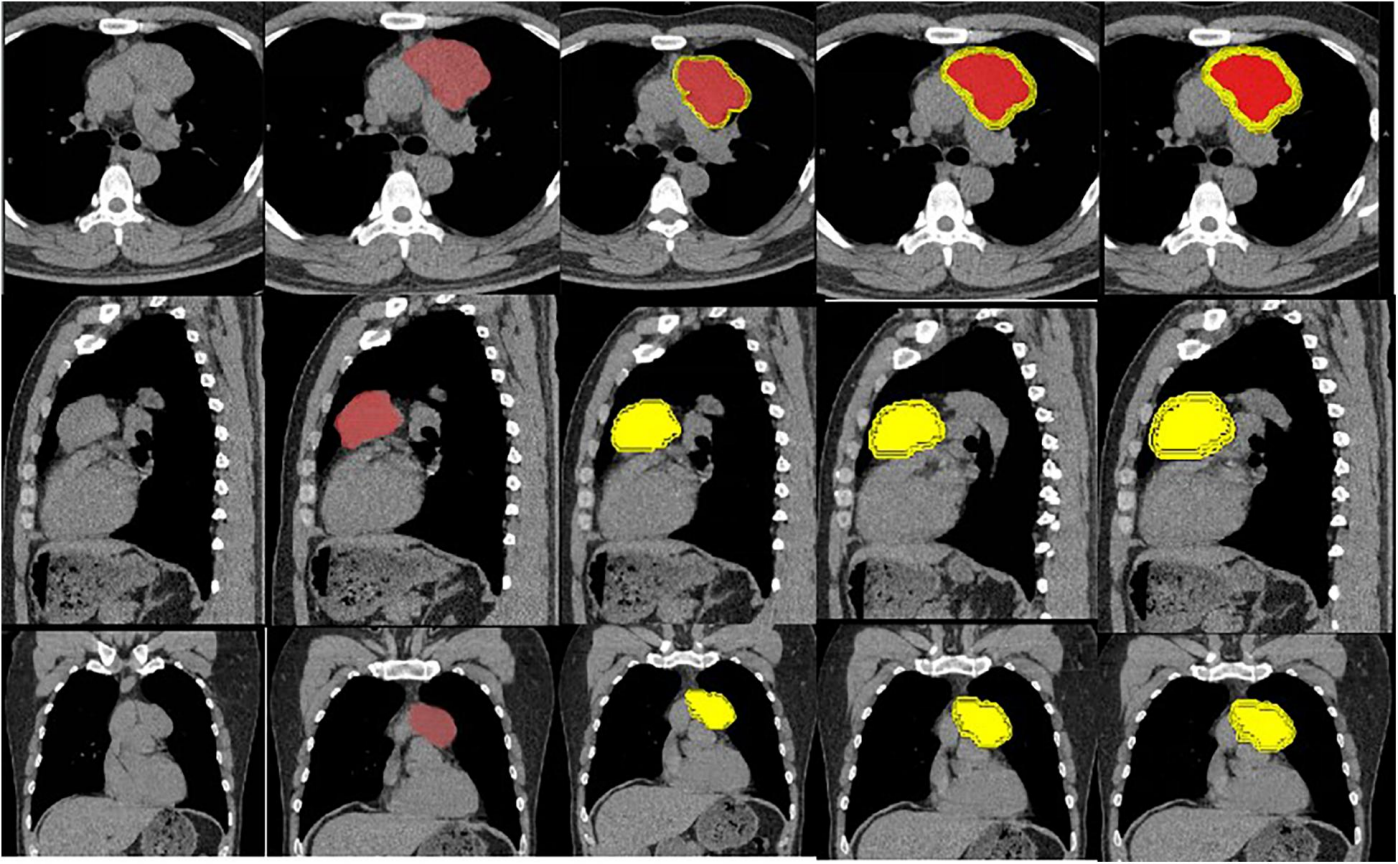
ROI delineation schematic for lesions.

All CT images underwent resampling and spatial normalisation to achieve a uniform voxel size of 1 ×  1 ×  1 mm^3^. Using ITK-SNAP software (8) (http://www.itksnap.org), regions of interest (ROIs) were delineated slice-by-slice on both non-contrast and contrast-enhanced axial CT images, with simultaneous validation and refinement in sagittal and coronal reformatted planes. All segmentation procedures were conducted using soft-tissue window settings. For each case, the primary tumour lesion was selected as the target region, with manual ROI delineation performed along the tumour boundary to ensure complete lesion inclusion while meticulously excluding adjacent non-tumoural structures including vessels, lymph nodes, and adipose tissue (Figure 3). The segmentation process was executed by a junior radiology resident with one year of experience, under the direct supervision of a senior radiologist possessing over ten years of expertise.

The resultant segmentation masks were subsequently processed using PHI-go AK software (GE Healthcare) to generate peri-tumoural regions through concentric expansion of the original tumour boundary by 2 mm, 4 mm, and 6 mm increments. Radiomic feature extraction was performed for both intratumoural and peritumoural regions, adhering strictly to the Image Biomarker Standardization Initiative (IBSI) guidelines. The extracted features encompassed the following categories: shape-based features (Shape), first-order statistical features (Firstorder), grey-level co-occurrence matrix features (GLCM), grey-level dependence matrix features (GLDM), grey-level run length matrix features (GLRLM), and grey-level size zone matrix features (GLSZM).

### 2.5 Batch effect assessment

To evaluate potential batch effects introduced by different CT scanners, we conducted principal component analysis (PCA) on the extracted radiomics features to visually assess clustering by scanner type. The analysis revealed no evident batch-related separation. For transparency, Supplementary Figure S1 illustrates the PCA visualization comparing features from both scanners, confirming minimal batch effect.

### 2.6 Model construction and evaluation

The dataset was partitioned into training and test sets using a 7:3 ratio. All preprocessing steps were performed strictly within the training set. The training set feature data underwent standardization and normalization. Subsequent feature dimensionality reduction and selection included: identifying and removing near-zero variance features; detecting highly collinear variables and eliminating redundant variables using a threshold of 0.8; performing univariate logistic regression analysis; and conducting LASSO regression analysis with 10-fold cross-validation. Predictive models were constructed for seven distinct regions: the tumour interior (T); the 2 mm peritumoural region (P2); the 4 mm peritumoural region (P4); the 6 mm peritumoural region (P6); the combined tumour interior and 2 mm peritumoural region (TP2); the combined tumour interior and 4 mm peritumoural region (TP4); and the combined tumour interior and 6 mm peritumoural region (TP6). Seven region-specific models were constructed for both non-contrast and contrast-enhanced images. Each of these was further developed using four machine learning algorithms—generalised linear model (GLM), random forest (RF), support vector machine (SVM), and naïve Bayes (NB)—yielding a total of 56 predictive models. Model hyperparameter optimisation was conducted using five-fold cross-validation within the training folds to ensure robust tuning and avoid data leakage.

A clinical model was developed using demographic and morphological variables, including sex, age, the largest and smallest diameters of the lesion on the maximal axial slice, lobulated margins, pleural contact, necrosis, and calcification. These features were selected based on their clinical relevance and prior evidence of association with disease characteristics. Prior to model construction, all features underwent preprocessing and correlation analysis to remove highly collinear variables. Significant predictors were identified through univariate analysis and subsequently incorporated into a multivariate logistic regression model. To enhance predictive performance, the clinical model was integrated with the optimal radiomics model, which was previously established through feature selection and machine learning algorithms. The combined model was constructed using a fusion strategy that incorporated both clinical and radiomics features into a single predictive framework via multivariate logistic regression. Model robustness and generalizability were assessed using five-fold cross-validation within the training cohort.

Each model's balanced accuracy, sensitivity, specificity, F1 score, kappa, precision and recall were systematically calculated. Receiver operating characteristic (ROC) and precision-recall (PR) curves were generated, with the corresponding area under the curve (AUC) values computed. Comprehensive confusion matrices were constructed for all models. The AUC and F1 score served as primary metrics for comparative performance assessment. Clinical utility was evaluated through decision curve analysis (DCA) to quantify net benefit.

SHAP (Shapley Additive Explanations) analysis was utilised to quantify individual feature contributions. The Voxel Based Feature Extract module within the PHI-go AK software enabled projection of the feature value matrix onto corresponding voxels of the source CT images, producing semi-transparent colour overlays to visualise the spatial distribution patterns of radiomic features.

### 2.7 Statistical analysis

Continuous variables, expressed as mean ± standard deviation, were compared between groups using the independent samples t-test. The chi-square test was employed to analyse differences in proportions for categorical variables (sex, clinical symptoms and comorbidities) between groups, with Fisher's exact test applied when any category contained fewer than five observations. Interobserver agreement in image quality assessment was evaluated using kappa statistics. DeLong's test was used to compare differences in the area under the curve (AUC) between predictive models. A two-sided *p*-value < 0.05 was considered statistically significant. All statistical analyses were performed using the following software packages: SPSS version 22.0 (IBM), R version 4.5.0 (https://www.rstudio.com), and Python version 3.7 (https://www.python.org).t

## 3 Results

### 3.1 Image quality assessment

The subjective image quality assessments provided by the two radiologists demonstrated moderate agreement, with a Cohen's kappa value of 0.603 (*p* < 0.01). In 99.34% of cases (151/152), the image quality satisfied the inclusion criteria, as both evaluators assigned scores of 4 or higher (Table 2).

**Table 2:** Interobserver agreement in image quality assessment.

Score	Radiologist A	Radiologist B
5	133	134
4	18	17
3	1	1
2	0	0
1	0	0

### 3.2 Patient clinical information

From the initial cohort of 218 eligible patients, 151 were ultimately enrolled in the study. No statistically significant differences (*p* > 0.05) were observed between the non-capsular invasion and capsular invasion groups regarding gender distribution, age, clinical presentation, or comorbid conditions (Table 3).

**Table 3:** Clinical characteristics of patients.

Characteristics	Non-invasion group (*n* = 53)	Invasion group (*n* = 98)	Statistical test	*p*-value
Male	30 (56.66%)	50 (51.02%)	0.646	0.422
Female	23 (43.34%)	48 (48.98%)		
Age, years, mean ± SD	55.61 ± 13.17	54.83 ± 11.40	0.393	0.695
Clinical presentation, *n* (%)				
Asymptomatic	39 (73.58%)	57 (58.16%)	0.617	0.102
Chest pain/tightness	9 (16.89%)	28 (28.57%)	0.909	0.164
Cough	3 (5.66%)	7 (7.14%)	0.011	0.916
Myasthenia gravis	1 (1.87%)	4（4.08%)	/	0.653
Ptosis	1 (1.87%)	2（2.04%)	/	1.000
Comorbidities, *n* (%)				
None	35 (66.04%)	63 (64.29%)	0.764	0.850
Coronary artery disease	3 (5.66%)	3 (3.06%)	/	0.906
Diabetes mellitus	1 (1.87%)	3 (3.06%)	/	0.735
Hypertension	14 (26.42%)	29 (29.59%)	0.920	0.820

### 3.3 Model performance evaluation

#### 3.3.1 Model development

**Figure 4:**
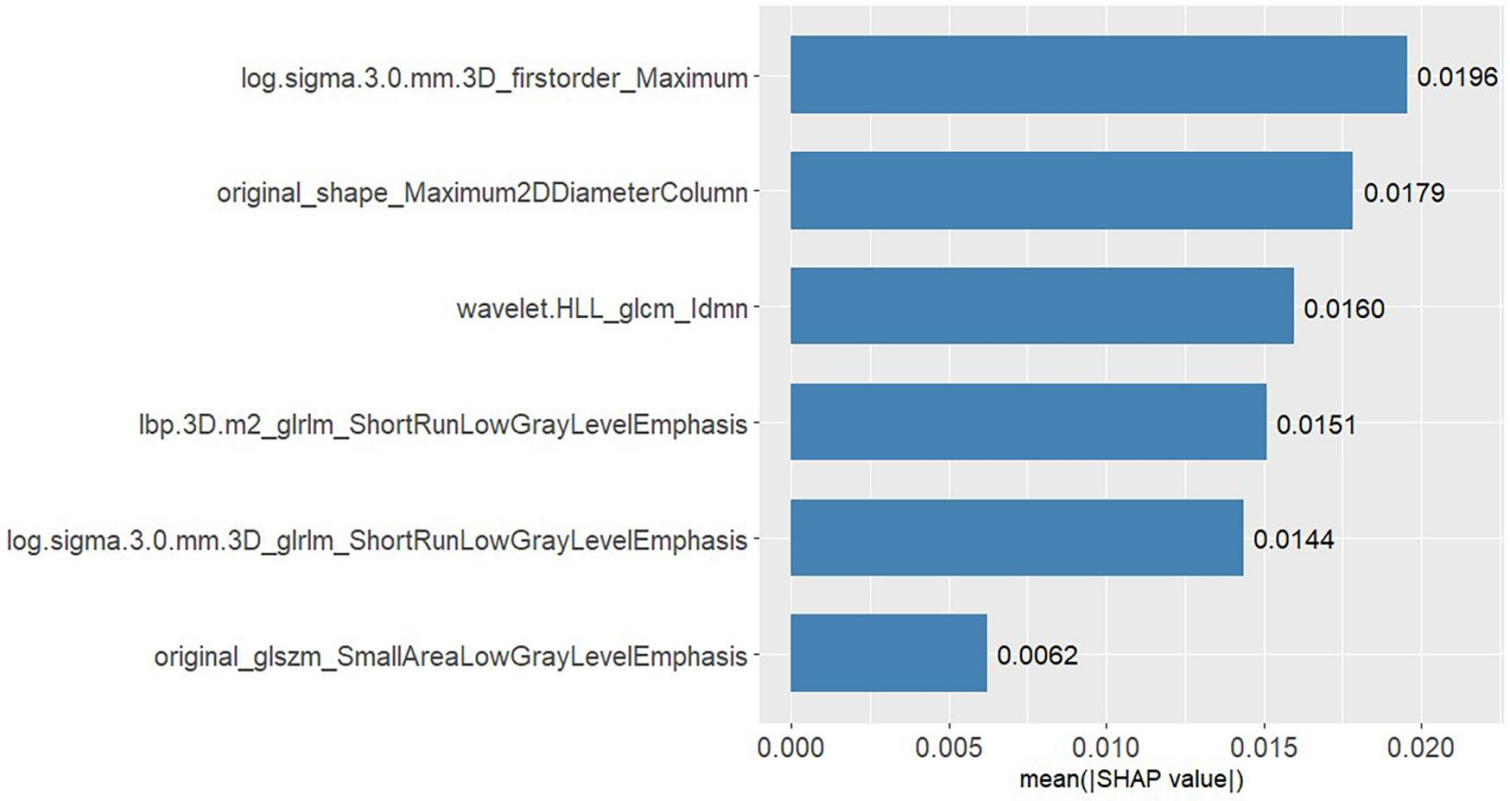
Feature selection results from the 4-mm peritumoural region (*Y*-axis: feature names; *X*-axis: importance scores).

Radiomic features were extracted from intratumoural, peritumoural, and combined intratumoural-peritumoural regions for all 151 patients included in the final analytical cohort. For each region, 1,316 features were systematically extracted. Through consistent feature selection protocols, the most clinically relevant features for predicting capsular invasion status were subsequently identified (Figure 4).

#### 3.3.2 Model performance evaluation

This study developed distinct machine learning prediction models using non-contrast and contrast-enhanced CT images, incorporating features derived from intratumoural regions and peritumoural regions at 2 mm, 4 mm, and 6 mm distances. Fifty-six individual radiomics models were constructed. For thymoma capsular invasion prediction, the contrast-enhanced CT model utilising the 4 mm peritumoural region achieved optimal performance in the test set, with AUC values of 0.862 (GLM), 0.888 (SVM), 0.813 (RF), and 0.446 (NB). While the RF model showed superior performance in the training set, the SVM model demonstrated the best performance among radiomics models in the test set, with the random forest model displaying overfitting tendencies in both training and test sets (Table 4). DeLong's test revealed significantly better predictive performance in contrast-enhanced models compared to non-contrast models. Among contrast-enhanced CT-based SVM models, each group was compared with the P4 group. The DeLong test results indicated no statistically significant difference (*P* > 0.05) in predictive performance (AUC) between the P4 model and most other enhanced SVM models (T, P2, TP4, TP6), while the P4 model showed significantly superior performance to the P6 and TP2 models (*P* < 0.05) (Table 5). Compared with the clinical model, the combined model that integrates clinical features with the P4 SVM-based radiomics approach demonstrated superior predictive performance (Table 6). The detailed results for all models are presented in the supplementary Tables (see Supplementary_Tables.xlxs).

**Table 4:** Performance metrics of the prediction models.

Regions	Algorithms	Non-contrast				Contrast-enhanced				DeLong*
		Training set		Test set		Training set		Test set		Test set
		AUC	CI	AUC	CI	AUC	CI	AUC	CI	*p*
T	GLM	0.713	(0.599–0.827)	0.655	(0.489–0.822)	0.742	(0.646–0.838)	0.881	(0.768–0.995)	0.031
	SVM	0.883	(0.812–0.955)	0.620	(0.443–0.796)	0.858	(0.769–0.946)	0.737	(0.591–0.884)	0.319
	RF	1.000	(1.000–1.000)	0.642	(0.459–0.824)	1.000	(1.000–1.000)	0.720	(0.567–0.872)	0.522
	NB	0.844	(0.762–0.927)	0.584	(0.396–0.772)	0.737	(0.639–0.834)	0.847	(0.721–0.973)	0.025
P2	GLM	0.656	(0.539–0.772)	0.448	(0.254–0.642)	0.814	(0.729–0.897)	0.744	(0.585–0.902)	0.066
	SVM	0.799	(0.704–0.894)	0.584	(0.404–0.764)	0.831	(0.739–0.923)	0.774	(0.631–0.917)	0.043
	RF	1.000	(1.000–1.000)	0.600	(0.406–0.795)	1.000	(1.000–1.000)	0.736	(0.575–0.897)	0.318
	NB	0.677	(0.568–0.786)	0.675	(0.495–0.854)	0.790	(0.702–0.878)	0.690	(0.515–0.865)	0.876
P4	GLM	0.705	(0.602–0.808)	0.754	(0.613–0.896)	0.816	(0.731–0.900)	0.862	(0.752–0.972)	0.008
	SVM	0.644	(0.524–0.764)	0.541	(0.345–0.737)	0.890	(0.823–0.956)	0.888	(0.792–0.983)	0.001
	RF	1	(1.000–1.000)	0.528	(0.359–0.698)	1.000	(1.000–1.000)	0.813	(0.680–0.945)	0.051
	NB	0.690	(0.584–0.797)	0.590	(0.417–0.764)	0.884	(0.818–0.951)	0.446	(0.238–0.654)	0.298
P6	GLM	0.698	(0.588–0.808)	0.571	(0.381–0.762)	0.723	(0.623–0.823)	0.754	(0.601–0.908)	0.003
	SVM	0.785	(0.688–0.882)	0.638	(0.456–0.820)	0.858	(0.769–0.948)	0.642	(0.467–0.817)	0.081
	RF	1	(1.000–1.000)	0.537	(0.365–0.709)	1.000	(1.000–1.000)	0.747	(0.583–0.910)	0.008
	NB	0.698	(0.588–0.807)	0.552	(0.354–0.749)	0.720	(0.620–0.819)	0.718	(0.555–0.881)	0.014
TP2	GLM	0.828	(0.741–0.914)	0.828	(0.701–0.954)	0.779	(0.689–0.869)	0.763	(0.615–0.911)	0.358
	SVM	0.878	(0.800–0.956)	0.823	(0.693–0.953)	0.889	(0.802–0.976)	0.774	(0.623–0.925)	0.421
	RF	1.000	(1.000–1.000)	0.683	(0.520–0.846)	1.000	(1.000–1.000)	0.758	(0.608–0.907)	0.459
	NB	0.830	(0.747–0.914)	0.821	(0.691–0.951)	0.856	(0.784–0.928)	0.685	(0.499–0.872)	0.138
TP4	GLM	0.796	(0.709–0.883)	0.776	(0.637–0.914)	0.716	(0.603–0.830)	0.642	(0.458–0.826)	0.185
	SVM	0.875	(0.801–0.948)	0.750	(0.603–0.897)	0.884	(0.806–0.962)	0.731	(0.582–0.880)	0.834
	RF	1.000	(1.000–1.000)	0.734	(0.578–0.890)	1.000	(1.000–1.000)	0.685	(0.525–0.846)	0.661
	NB	0.883	(0.820–0.946)	0.655	(0.474–0.836)	0.715	(0.608–0.823)	0.647	(0.449–0.844)	0.952
TP6	GLM	0.692	(0.574–0.810)	0.666	(0.473–0.859)	0.774	(0.678–0.870)	0.623	(0.441–0.805)	0.709
	SVM	0.780	(0.678–0.882)	0.662	(0.487–0.836)	0.881	(0.808–0.955)	0.769	(0.619–0.919)	0.323
	RF	1.000	(1.000–1.000)	0.431	(0.242–0.620)	1.000	(1.000–1.000)	0.710	(0.546–0.875)	0.052
	NB	0.785	(0.688–0.882)	0.614	(0.423–0.806)	0.762	(0.668–0.856)	0.649	(0.457–0.840)	0.800

T, intratumoural region; P2, peritumoural region (2 mm); P4, peritumoural region (4 mm); P6, peritumoural region (6 mm); TP2, combined intratumoural-peritumoural region (2 mm); TP4, combined intratumoural-peritumoural region (4 mm); TP6, combined intratumoural-peritumoural region (6 mm). DeLong, DeLong's test comparing the AUC between non-contrast and contrast-enhanced models in the test set.

**Table 5:** Performance comparison between contrast-enhanced SVM models and the P4 reference model (DeLong test).

Regions	P4	DeLong 95% CI
T	0.095	(−0.328–0.026)
P2	0.074	(−0.239–0.011)
P6	0.001	(−0.389–0.102)
TP2	0.048	(−0.227–0.001)
TP4	0.063	(−0.323–0.009)
TP6	0.118	(−0.267–0.030)

**Table 6:** Performance metrics of clinical model, combine model and the contrast-enhanced P4 SVM model in the test set.

Model	AUC	CI	Balanced accuracy	PR-AUC	Kappa	F1-score
Clinical model	0.818	(0.670–0.966)	0.799	0.840	0.568	0.875
Combine model	0.853	(0.735–0.972)	0.810	0.886	0.633	0.852
P4 SVM model	0.888	(0.792–0.983)	0.789	0.903	0.570	0.842

**Figure 5:**
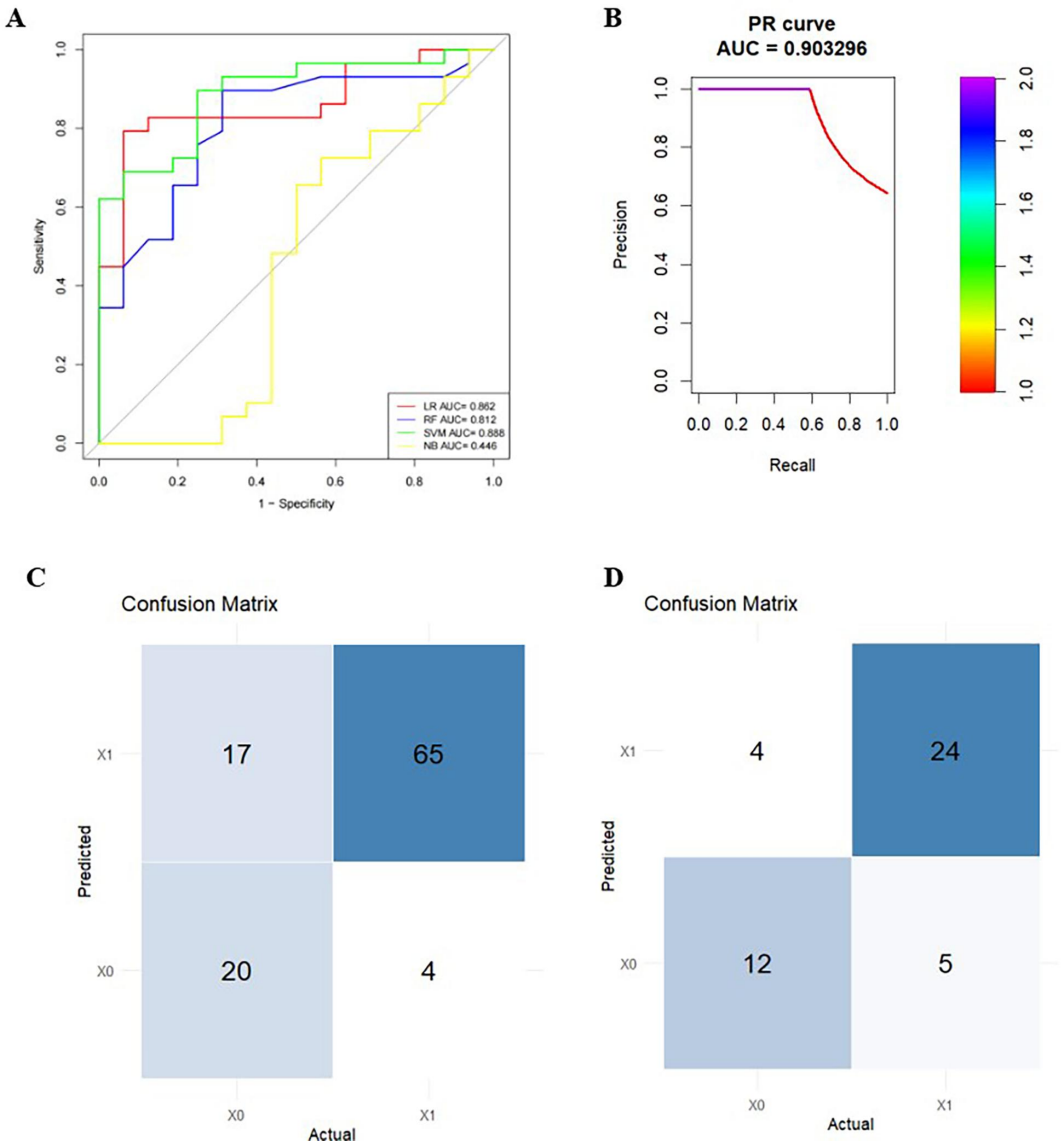
**(A)** receiver operating characteristic (ROC) curves of the four machine learning algorithms in the contrast-enhanced P4 test set. **(B)** Precision-recall curves. **(C, D)** Confusion matrices for the training and test sets.

**Figure 6:**
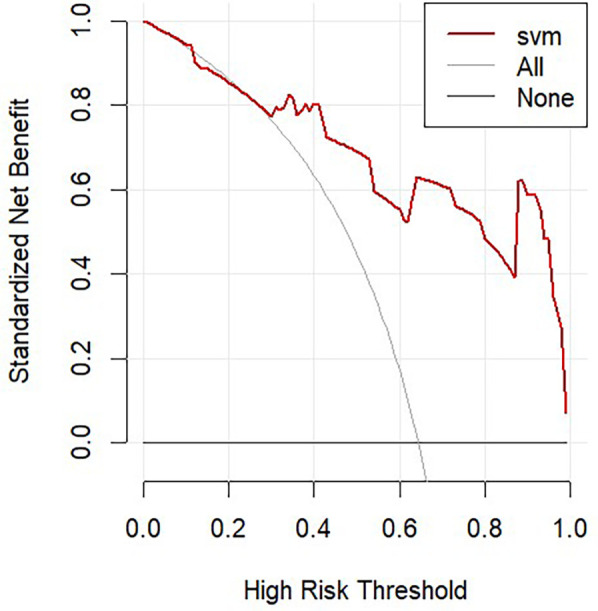
Decision curve analysis (DCA) for clinical assessment.

The calibration curves for the training and test sets indicated good calibration performance of the SVM model (Figure 5B). The confusion matrices demonstrated satisfactory classification performance of the prediction model (Figures 5C,D). Decision curve analysis revealed net clinical benefit of the model in the validation set (Figure 6).

#### 3.3.3 Model visualisation

**Figure 7:**
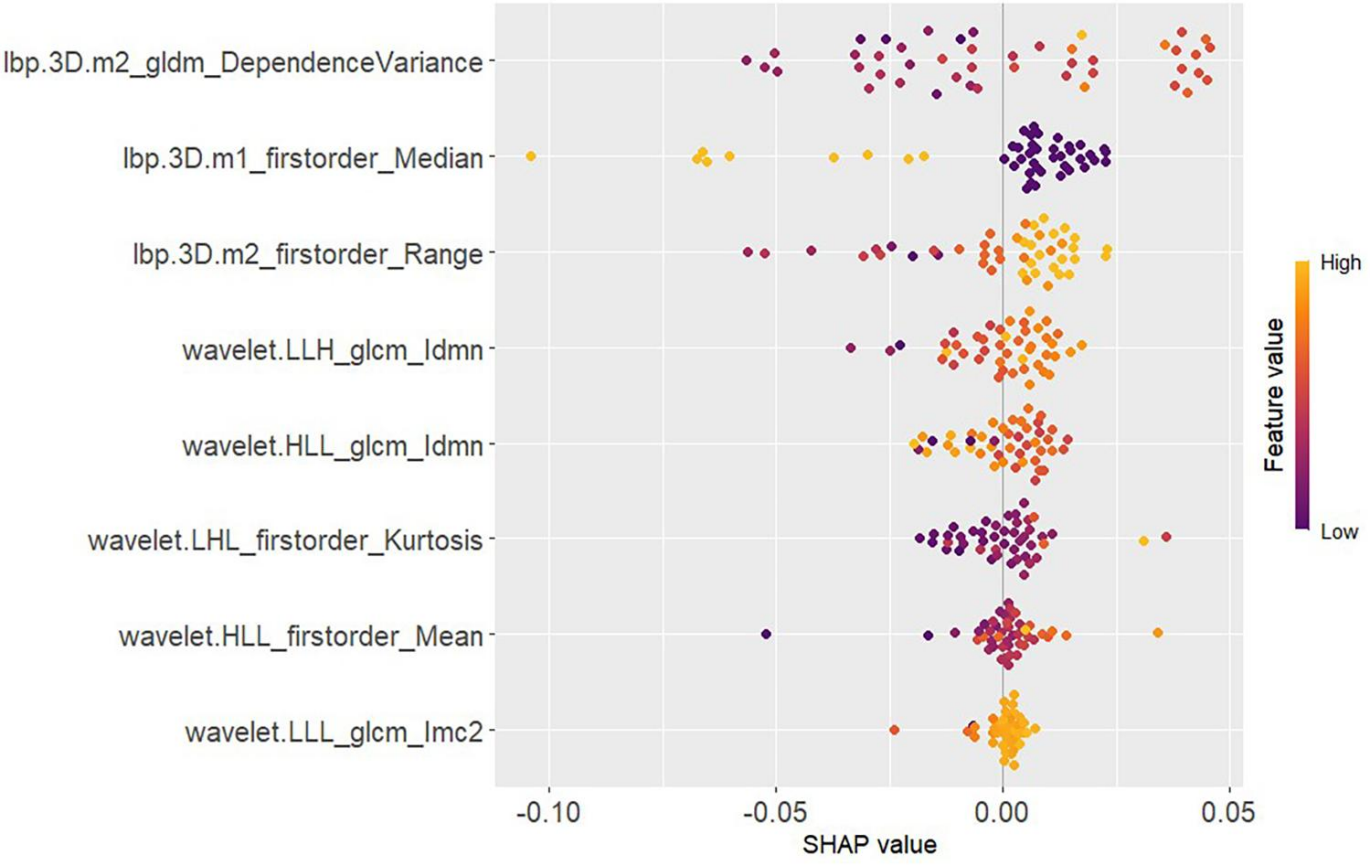
SHAP values in the test set.

**Figure 8:**
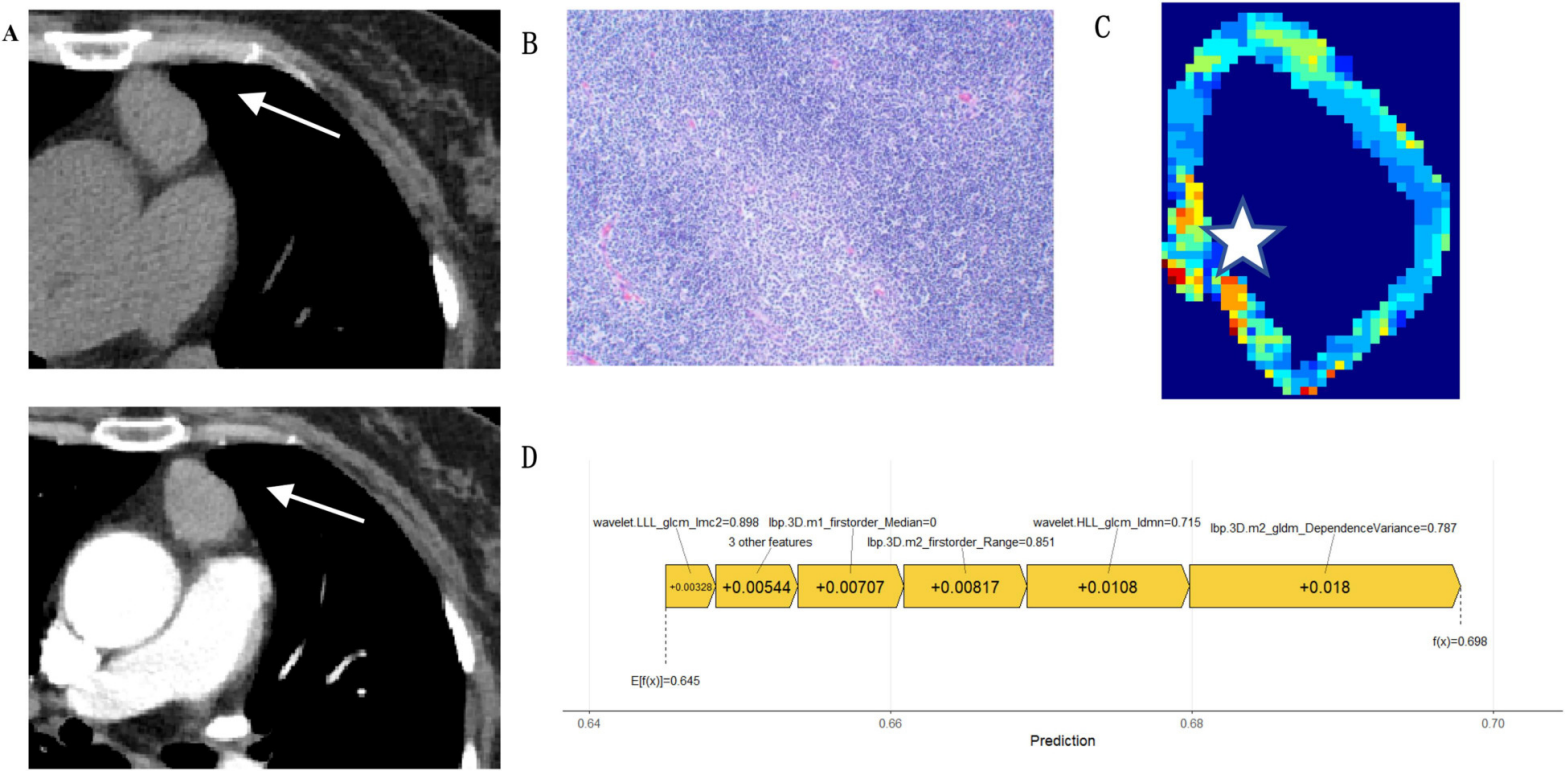
Case 1: A 62-year-old male. **(A)** Non-contrast CT demonstrates a soft-tissue density lesion in the left anterosuperior mediastinum (white arrow), showing mild enhancement on contrast-enhanced imaging. **(B)** Histopathological examination confirmed type A thymoma with capsular invasion (haematoxylin and eosin stain; × 100 magnification). **(C)** The feature mapping heatmap visualisation reveals red areas representing the weighted feature value distribution predictive of capsular invasion (white star). **(D)** SHAP analysis demonstrates the relative contribution of individual features.

**Figure 9:**
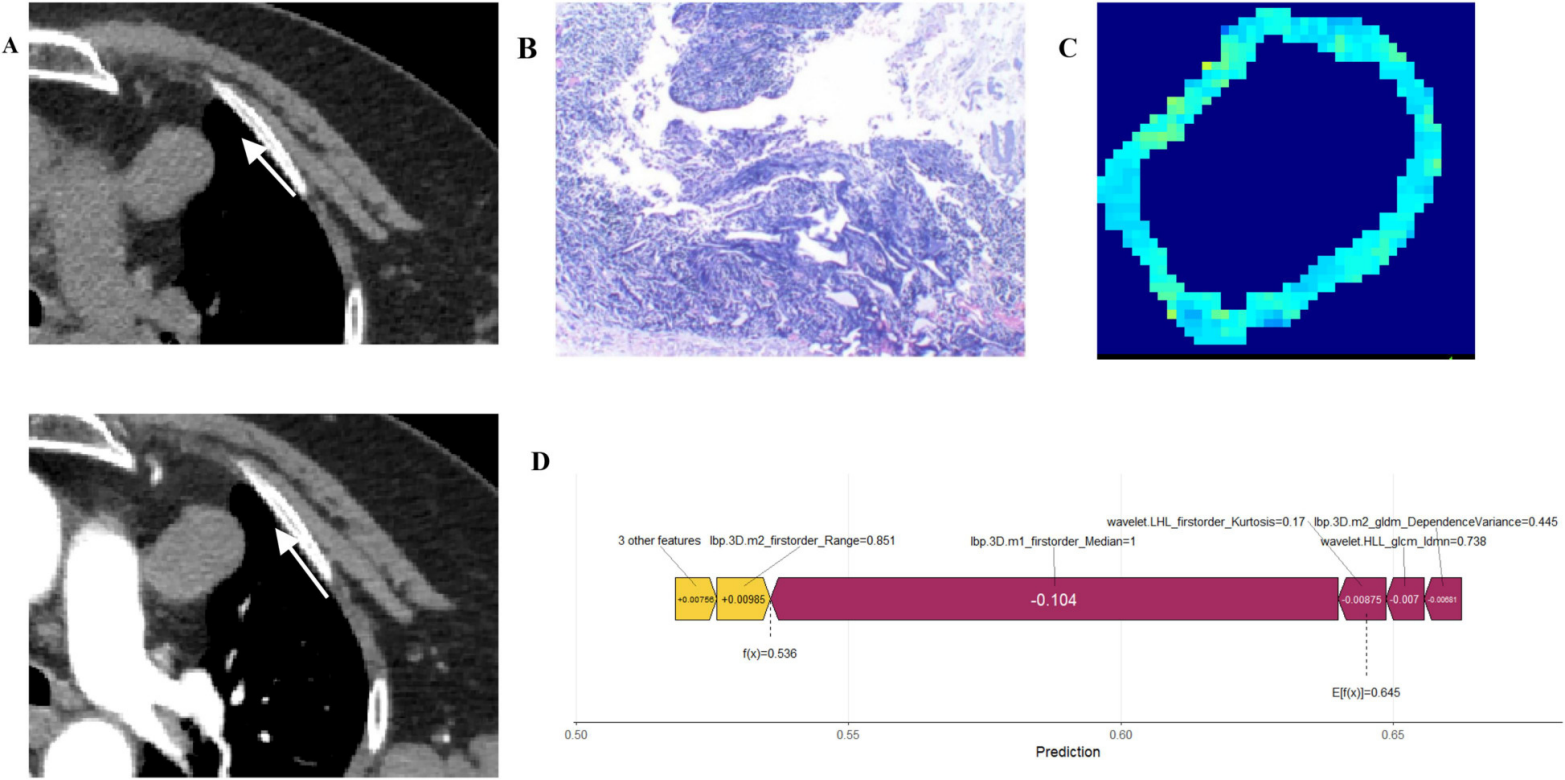
Case 2: A 67-year-old male.

This study employed SHAP methodology to interpret model predictions, elucidating the contribution of individual features to decision-making. The SHAP value scatter plot displays features against their corresponding SHAP values for patients in the test set, with clear demarcation of positive and negative predictive influences (Figure 7). Detailed descriptions of these features, along with their biological interpretations, are provided in Supplementary Table S1 (see Supplementary_Material.docx). Individual SHAP force plots visualise capsular invasion predictions in thymoma patients, presenting two representative correctly predicted cases (Figures 8, 9).

A. Non-contrast CT demonstrates a soft-tissue density mass in the left anterosuperior mediastinum, exhibiting mild enhancement on contrast-enhanced imaging with shallow lobulation at the lesion margin (white arrow).
B. Histopathological examination revealed type A thymoma with no evidence of capsular invasion (haematoxylin and eosin stain; × 100 magnification).
C. The feature mapping heatmap visualisation displays blue regions representing the weighted feature value distribution predictive of absent capsular invasion.
D. SHAP analysis demonstrates the relative feature contributions.

## 4 Discussion

This study constructed multiple prediction models using CT radiomic features and identified the most robust model as the SVM algorithm based on the 4 mm peritumoural region of contrast-enhanced CT scans, achieving AUC values of 0.890 in the training set and 0.888 in the validation set, indicating high diagnostic efficacy for predicting capsular invasion in thymoma.

Thymomas are highly heterogeneous (9), and capsular invasion may occur even in small tumors. In thymoma management, early prediction of capsular invasion helps clinicians select appropriate surgical approaches and determine whether postoperative adjuvant therapy is required, which directly affects treatment efficacy and patients' quality of life.

Contrast-enhanced CT represents the optimal imaging modality for preoperative thymoma evaluation (10). While non-contrast CT demonstrates utility for initial screening through tissue density differentiation, contrast-enhanced CT provides superior visualisation of tumour vascularity and tissue perfusion via contrast medium administration (11). This study revealed consistently better predictive performance with contrast-enhanced CT-based models compared to non-contrast CT models, reflecting the increased invasive potential and neovascularisation associated with capsular invasion in thymomas. These findings further indicate that contrast-enhanced CT-derived radiomic features contain more biologically relevant information, permitting machine learning models to more accurately identify subtle pathological alterations indicative of capsular invasion.

During radiomics model construction, multiple machine learning algorithms were implemented, comprising random forest (RF) and support vector machine (SVM). While the RF model demonstrated optimal performance in the training set, its test set performance revealed overfitting tendencies, reflecting generalisability constraints. These findings highlight the criticality of judicious feature selection and algorithm choice for optimising model performance (12). DeLong's test results showed no statistically significant difference (*p* > 0.05) in predictive performance (AUC) between the P4 model and intratumoural model (T), indicating that both intratumoural heterogeneity and peritumoural microenvironmental characteristics contribute substantially to thymoma invasiveness prediction post capsular invasion.

The peritumoural region harbours significant information about tumour heterogeneity. While existing research has predominantly examined intratumoural microenvironment heterogeneity, the biological relevance of peritumoural microenvironment alterations has frequently been neglected (13). Clinically, although most tumour characteristics are evaluated within the tumour proper, residual peritumoural tissue persists following initial surgical resection and may influence local recurrence patterns (14). Wang et al. (15) established an MRI-based radiomics model incorporating both intratumoural and peritumoural features to predict extra-pelvic peritoneal metastasis in epithelial ovarian cancer, demonstrating superior performance of peritumoural and combined models over the intratumoural model in internal validation (AUC 0.786 and 0.832 vs. 0.652; *p* = 0.007 and *p* < 0.001, respectively). Similarly, Zhang et al. (16) successfully integrated perithymic radiological and semantic features for histopathological risk stratification of thymic epithelial tumours (AUC 0.857), further substantiating the importance of peritumoural assessment in capturing global tumour spatial heterogeneity.

This study investigated peritumoural models at 2 mm, 4 mm and 6 mm distances from the tumour boundary. Following feature selection, six peritumoural features were identified as most clinically relevant and subsequently integrated into the predictive models. The 4 mm peritumoural region demonstrated optimal performance for capsular invasion prediction, establishing the diagnostic superiority of peritumoural feature analysis over conventional intratumoural assessment in thymoma. Peritumoural radiomics provides unique capabilities for capturing high-order boundary characteristics, particularly in thymomas with irregular or poorly defined margins (17). Among the high-weight features, *original_shape_Maximum2DDiameterColumn* showed strongest direct correlation with capsular invasion, quantifying morphological attributes where increased tumour diameter elevates the likelihood of capsular contact and penetration. Complementarily, reduced grey-level co-occurrence matrix uniformity values (wavelet.HLL_glcm_idmn) reflected marked microenvironmental heterogeneity. Collectively, these radiomic signatures serve as imaging biomarkers of tumour aggressiveness, encapsulating textural patterns indicative of heterogeneous growth and destructive invasion potential, thereby elucidating the invasive biological behaviour characteristic of thymomas.

Model interpretability is fundamental for clinical deployment. This study established predictive models with visualization of both final outputs and the complete feature selection workflow, objectively demonstrating model accuracy while presenting all associated outcomes.

The relatively small study cohort reflects the low incidence of thymoma. Being a retrospective single-centre investigation, this study may be subject to selection bias. Future research should include multicentre datasets with external validation to thoroughly evaluate the model's efficacy and robustness. Manual segmentation of regions of interest may introduce observer bias, highlighting the need for automated segmentation methods in subsequent studies to improve the precision and reproducibility of radiomic feature extraction.

In summary, this study has established a CT-based radiomics model for predicting thymoma capsular invasion. This non-invasive method demonstrates satisfactory predictive accuracy and stability, providing clinicians with valuable preoperative references for treatment planning.

## Data Availability

The raw data supporting the conclusions of this article will be made available by the authors, without undue reservation.
