# Supplementary material for: Construction of a radiomics model based on CT imaging for predicting capsular invasion in thymomas
Source: Front Radiol. 2025 Dec 12;5:1707488. doi: 10.3389/fradi.2025.1707488 (PMC12740908; doi:10.3389/fradi.2025.1707488)
Supplement: Supplementary file 1 [file Table1.docx]

**Figure S1: The PCA visualization comparing features from both scanners**

**
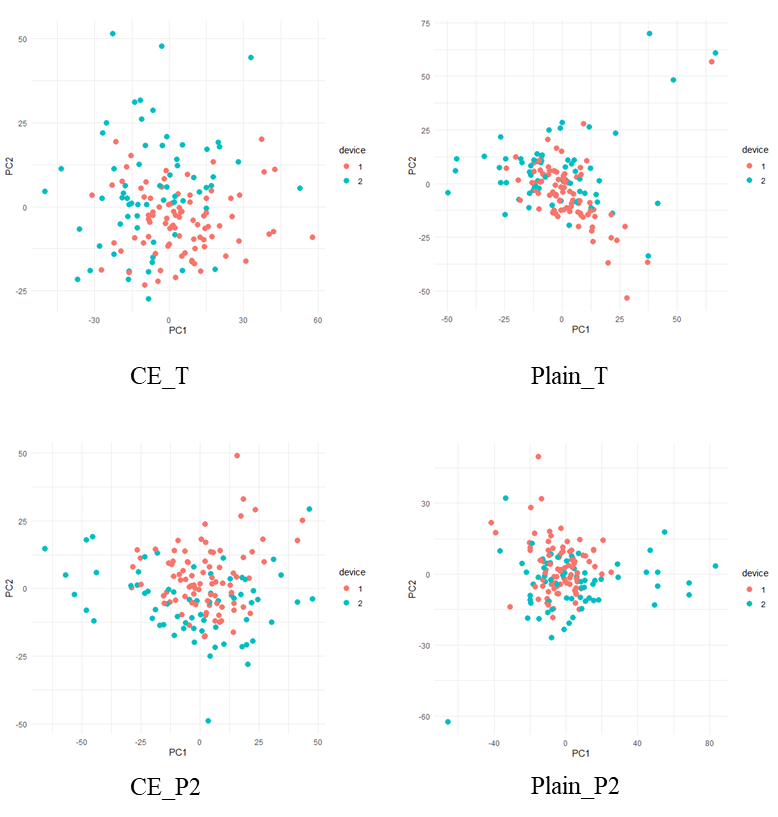
**
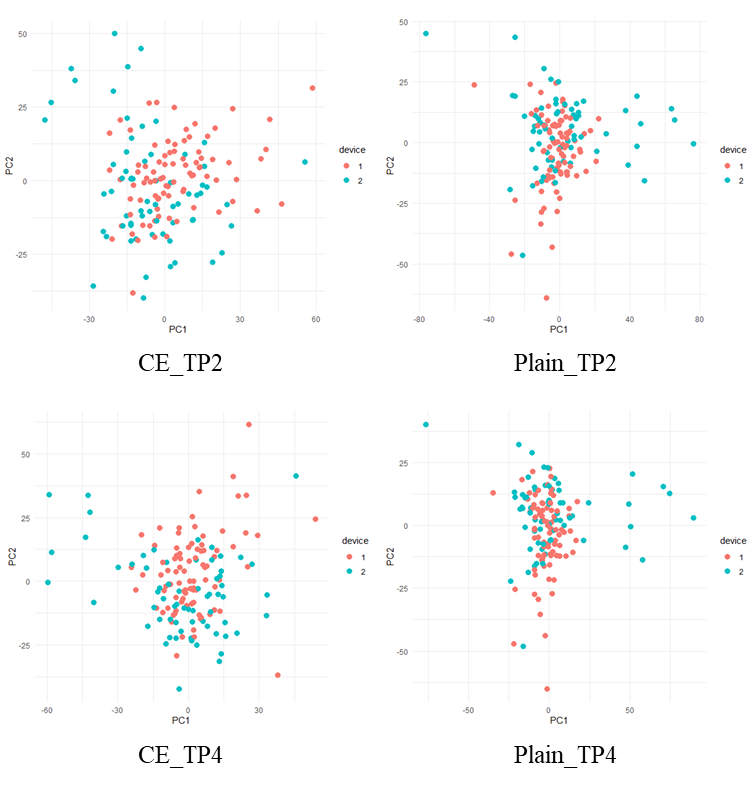


**
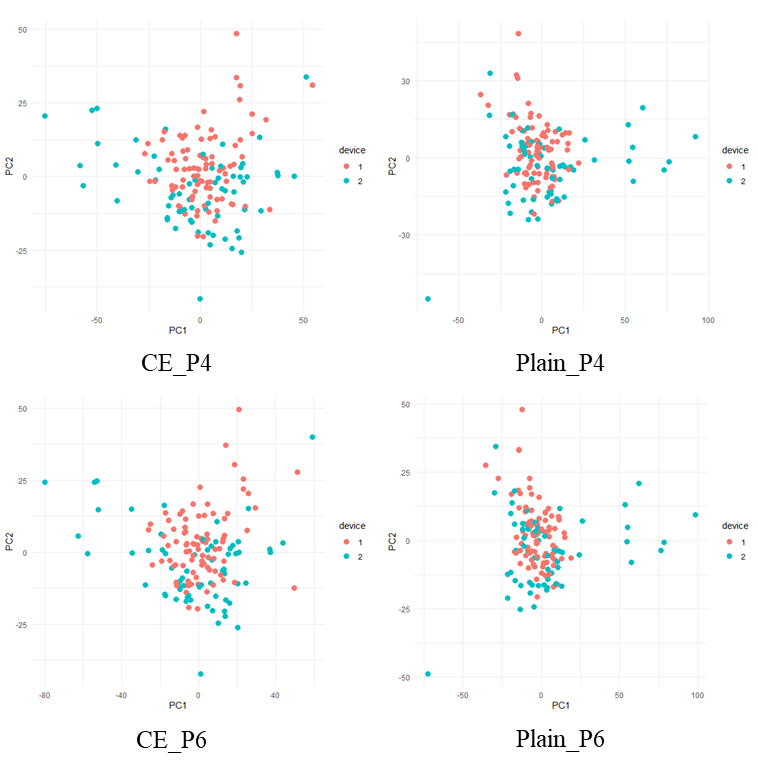
**
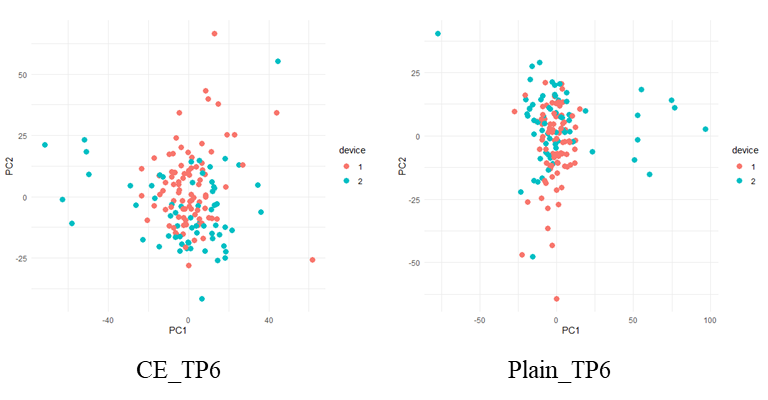


**Table S1: Top Radiomic Features and Biological Interpretations**

| **Feature** | **Description** | **Biological Interpretation** |
| --- | --- | --- |
| **lbp.3D.m2_gldm_DependenceVariance** | Variance of gray-level dependence in GLDM | Reflects structural complexity and heterogeneity; higher values may indicate aggressive tumor microenvironment. |
| **lbp.3D.m1_firstorder_Median** | Median intensity value of LBP-based texture | Represents overall signal intensity; may correlate with tumor density or cellular composition. |
| **lbp.3D.m2_firstorder_Range** | Range of intensity values (max–min) | Large range suggests internal heterogeneity, necrosis, or mixed tissue components. |
| **wavelet.LLH_glcm_Idmn** | Inverse Difference Moment Normalized from GLCM (LLH wavelet) | Higher values indicate texture uniformity; lower values suggest heterogeneity and irregular structure. |
| **wavelet.HLL_glcm_Idmn** | Same metric as above, computed on HLL wavelet decomposition | Captures uniformity at different spatial scales; may relate to tumor grade or microenvironment variability. |
| **wavelet.LHL_firstorder_Kurtosis** | Kurtosis of intensity distribution | High kurtosis indicates concentrated intensity distribution; low kurtosis suggests heterogeneity. |
| **wavelet.HLL_firstorder_Mean** | Mean intensity value | May correlate with tissue density, vascularity, or cellular abundance. |
| **wavelet.LLL_glcm_Imc2** | Informational Measure of Correlation 2 from GLCM | Reflects texture complexity; higher values indicate heterogeneous microenvironment and potential aggressiveness. |
